# Acute effects of lactate infusion on metabolism, AD biomarkers, and cognition; the LEAN study

**DOI:** 10.1002/alz70856_106240

**Published:** 2026-01-08

**Authors:** Riley E Kemna, Paul J Kueck, Anneka Blankenship, Casey S. John, Chelsea N Johnson, Zachary D. Green, John P Thyfault, Jonathan D Mahnken, Benjamin Miller, Jill K Morris

**Affiliations:** ^1^ University of Kansas Alzheimer's Disease Research Center, Fairway, KS, USA; ^2^ University of Kansas Medical Center, Kansas City, KS, USA; ^3^ Oklahoma Medical Research Foundation, Oklahoma City, OK, USA

## Abstract

**Background:**

Cerebral hypometabolism is a hallmark of Alzheimer's Disease (AD) and it is possible that alternative fuel substrates such as lactate could be beneficial in AD. However, how efficiently lactate is metabolized in AD individuals compared to cognitively healthy (CH) older adults has never been characterized.

**Methods:**

CH (*n* = 12) and AD (*n* = 12) older adults were enrolled into the Lactate for Energy and Neurocognition (LEAN) trial at the KU ADRC (NCT05207397). Subjects underwent a single study visit “lactate clamp”, where they received stable infusions of D_2_‐glucose, [3‐^13^C] sodium lactate, and a variable infusion of unlabeled sodium lactate for 120 minutes to achieve 4mM blood lactate. Cognitive tests (NIH toolbox) were administered prior to infusion and at minute 90, during steady state. Breath and blood sampling were performed at 0, 60, 75, 90, and 120 minutes to calculate lactate metabolism. AD blood biomarkers were assessed at 0 and 120 minutes in EDTA plasma. Brain‐derived tau (BD‐tau), pTau217, pTau181, total tau, glial fibrillary acidic protein (GFAP), neurofilament light‐chain (NfL), and Brain Derived Neurotrophic Factor (BDNF) were analyzed by Simoa‐HDX (Quanterix). β‐amyloid 42 and 40 were analyzed using Lumipulse (Fujirebio). We measured creatinine (RayBiotech) and hematocrit (Immunostics, Inc) to assess change in kidney function and blood volume.

**Results:**

AD subjects oxidized lactate as well as CH subjects (*p* = 0.988). Processing speed (*p* <0.001) and global cognition (*p* <0.001) were improved after infusion. Interestingly, we observed reductions in plasma BD‐tau (*p* <0.001), pTau217 (*p* <0.001), pTau181 (*p* <0.001), GFAP (*p* <0.001), and NfL (*p* <0.001) in both AD and CH groups after lactate infusion. Total tau and BDNF levels were unchanged by infusion (*p* = 0.133 and 0.182). Pre/post change in plasma volume and eGFR were 4% and 16.9%, respectively.

**Conclusion:**

Our objective was to characterize whole‐body lactate metabolism in AD, an understand how lactate might affect cognition. We found that lactate infusion reduced BD‐tau, phosphotau species, GFAP, and NfL which could be due to systemic or brain‐based changes in proteostasis. Biomarker changes were not explained by change in plasma volume or kidney function, and only brain‐specific biomarkers were affected. Additional analyses are planned to investigate the relationship of lactate and perturbations in AD biomarkers.

